# Prime Editing Corrects Multiple Mutations in the RSRSP Region of the *RBM20* Gene Using a Single Prime Editing Guide RNA

**DOI:** 10.31662/jmaj.2025-0004

**Published:** 2025-03-21

**Authors:** Takahiko Nishiyama

**Affiliations:** 1 Department of Cardiology, Keio University School of Medicine, Tokyo, Japan

**Keywords:** gene editing, prime editing, dilated cardiomyopathy, RBM20

Dilated cardiomyopathy (DCM) is a severe cardiac condition characterized by ventricular dilation and impaired systolic function, often progressing to heart failure. Mutations in the RNA-binding motif protein 20 (*RBM20*) gene are identified in 2%-6% of patients with DCM and represent a significant cause of familial DCM ^(1), (2)^. The RBM20 protein plays a critical role in regulating the splicing of titin (*TTN*) and other genes essential for cardiac function ^(3)^. Mutations in the RSRSP (AA634-AA638) region of *RBM20* gene cause the mislocalization of the RBM20 protein into cytoplasm, contributing to DCM pathogenesis ^(4), (5), (6)^. Clustered regularly interspaced short palindromic repeats and CRISPR-associated protein 9 (CRISPR/Cas9) operates by inducing double-strand breaks in DNA, which are subsequently repaired by the natural repair mechanisms ^(7)^. Although this approach is highly effective in certain applications, it carries the risk of unintended consequences, such as insertions, deletions (indels), or other mutations at the target site or even at off-target locations. These unintended genetic alterations may lead to harmful effects, especially in complex and tightly regulated tissues such as the heart. As a result, there is an increasing need for gene-editing methods that are more precise and less disruptive. Prime editing (PE), a novel and versatile CRISPR-based technique, offers a promising solution to this problem ^(8)^. Instead of creating double-strand breaks, PE uses a fusion protein comprising a catalytically impaired Cas9 endonuclease and a reverse transcriptase. This complex is guided to the target DNA site by a PE guide RNA (pegRNA). Once there, the reverse transcriptase uses the pegRNA as a template to make precise edits to the DNA sequence. This method enables targeted indels and base conversions with high precision and minimal off-target effects.

In previous studies, PE using engineered pegRNA (epegRNA ^(9)^) and PEmax significantly corrected the R636S mutation in induced pluripotent stem cells ^(10)^. This highlights the potential of PE to correct specific mutations accurately. Although PE has the potential to correct multiple mutations in the hotspot region with a single designed pegRNA, this capability has not been thoroughly investigated. To evaluate whether PE can successfully correct multiple mutations, the same system was used in HEK293 cells with other mutations in the *RBM20* gene. To establish mutant strains and evaluate gene-editing efficiency in HEK293 cells, RBM20 variants (R634W and S635A) were generated by constructing vectors containing synthesized mutant sequences. These sequences were cloned into plasmids and transfected into HEK293 cells, followed by DNA extraction and verification through Sanger sequencing. PE was performed using the PE3b system, incorporating plasmids and pegRNA sequences designed from DNA oligos. Moreover, epegRNA was also included to enhance editing efficiency. The successful integration of these genetic modifications was confirmed through Sanger sequencing. Specifically, the results revealed successful gene editing for both the R634W and S635A mutations. After transfection with the PE system, the editing efficiencies were 53% and 55% for the R634W and S635A mutations, respectively (Figure 1). Sanger sequencing analysis further verified precise editing at the targeted sites, with minimal indels or unintended mutations.

**Figure 1.**
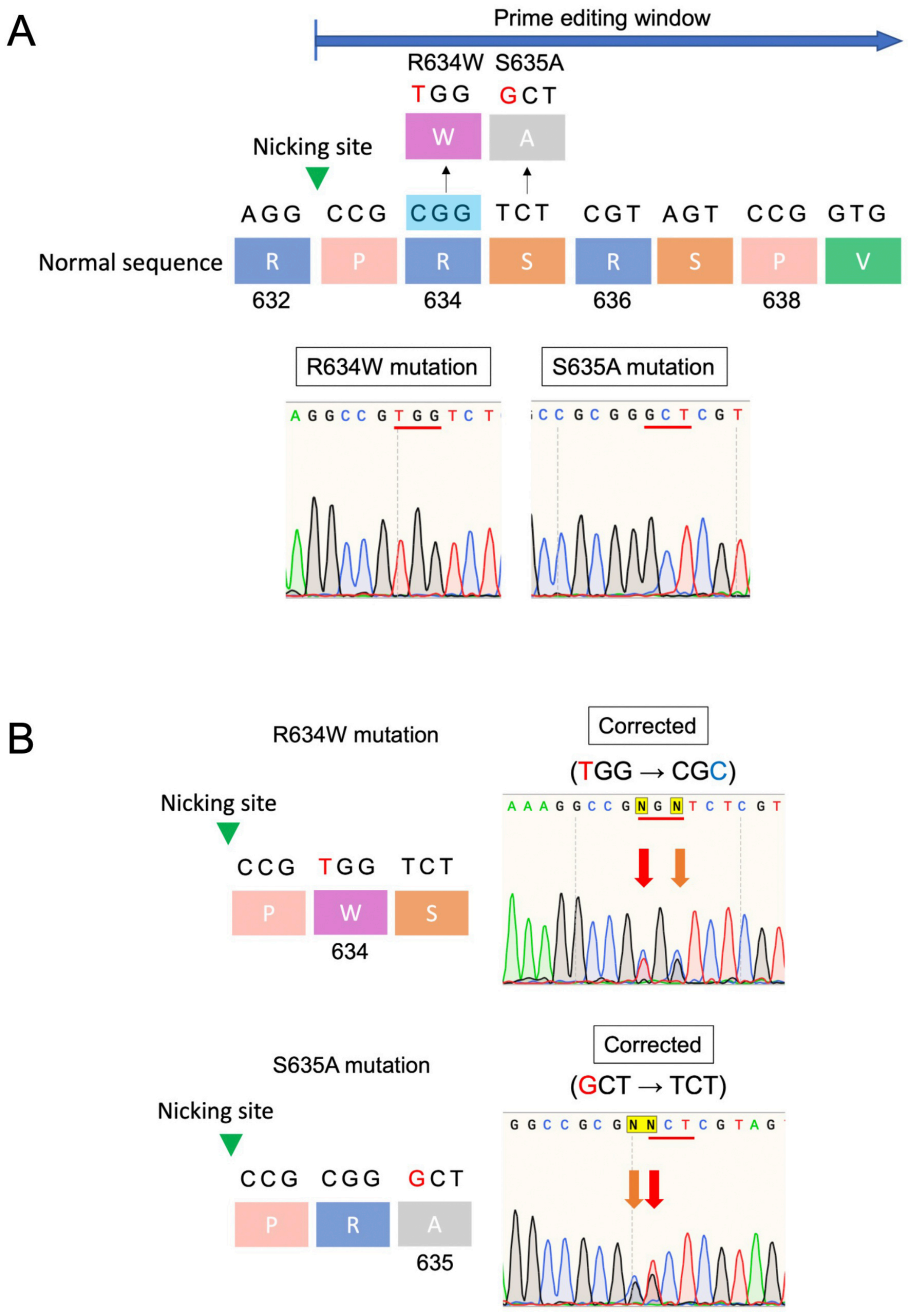
A: HEK293 cells expressing the RBM20 variants (R634W and S635A) were generated separately. The green arrowhead indicates the nicking site, whereas the NGG (blue box) denotes the PAM sequence for SpCas9 used in PE. B: PE corrected multiple mutations within the RSRSP region, including R634W and S635A. The R634W mutation (TGG) was corrected to CGC, encoding arginine (R), whereas the S635A mutation (GCT) was corrected to TCT, encoding serine (S). PE: prime editing.

PE represents a significant advancement over traditional gene-editing techniques for correcting *RBM20* mutations. Its ability to accurately target and correct multiple hotspot mutations in the *RBM20* gene without inducing double-strand breaks or causing substantial off-target effects highlights its potential as a promising therapeutic strategy for DCM. The high fidelity and efficiency of this method also pave the way for its application in other genetic disorders characterized by similar mutation hotspots. By addressing the cause of the disease at the genetic level, PE offers the potential for a durable solution that surpasses the symptomatic relief provided by current treatments. Compared with traditional gene-editing techniques, PE provides several key advantages: its precision eliminates the need for double-strand breaks, thereby minimizing the risk of unintended mutations and other adverse effects. Moreover, its versatility enables the correction of a broad range of genetic mutations, making it applicable to numerous genetic disorders beyond DCM.

This study indicates the successful correction of multiple mutations in the RSRSP region of *RBM20* gene using PE, underscoring its potential as a therapeutic strategy for genetic cardiomyopathies. Future work should focus on in vivo studies to evaluate the long-term safety and efficacy of PE in animal models of RBM20-associated DCM. In addition, developing delivery systems for PE components to heart tissue will be crucial for translating this technology into clinical therapies. The ability of PE to address multiple mutations with a single intervention could revolutionize the treatment of genetic disorders, offering hope for patients with DCM and potentially other conditions caused by similar genetic mutations. However, one of the major challenges in developing gene-editing therapies is ensuring the efficient and safe delivery of editing components to target tissues ^(11)^. For DCM, this involves delivering the PE machinery specifically to cardiomyocytes. Various delivery methods, such as viral vectors, lipid nanoparticles, and other novel delivery systems, are being explored to achieve this goal ^(12), (13)^. Each method has its advantages and limitations, and finding the optimal delivery system will be crucial for the success of PE-based therapies ^(14)^. Furthermore, long-term studies are necessary to assess the durability of PE edits. It is important to determine whether the corrected cells maintain their function over time and to evaluate any potential long-term risks associated with gene editing. Addressing these challenges will be essential to ensure PE provides a safe, effective, and durable treatment for DCM and other genetic disorders.

In conclusion, PE represents a significant advancement in gene therapy, offering precision and versatility for correcting mutations in the *RBM20* gene. The successful correction of multiple mutations in this study highlights the potential of PE to address a broad range of genetic disorders.

To translate this technology into clinical applications, further research must focus on developing efficient delivery systems and ensuring long-term safety. PE has the potential to revolutionize the treatment of genetic diseases, providing new hope to patients and paving the way for a new era of precision medicine.

## Article Information

This article is based on the study, which received the Medical Research Encouragement Prize of The Japan Medical Association in 2023.

### Conflicts of Interest

None

### Sources of Funding

This study was funded by a Grant-in-Aid for Scientific Research (Grant Number 17K09585) and by unrestricted research grants from Japan Heart Foundation Research Grant for Dilated Cardiomyopathy, Fukuda Foundation for Medical Technology, SENSHIN Medical Research Foundation, and The Mochida Memorial Foundation for Medical and Pharmaceutical Research.

### Acknowledgement

The author thanks the Center for Integrated Medical Research at Keio University for its invaluable support and guidance throughout this work.

### Approval by Institutional Review Board (IRB)

All protocols in this study were approved by the Genetic Modification Safety Committee, Keio University School of Medicine (D2024-002).
